# Volatiles from male honeydew excretions attract conspecific male spotted lanternflies, *Lycorma delicatula* (Hemiptera: Fulgoridae)

**DOI:** 10.3389/finsc.2022.982965

**Published:** 2022-09-27

**Authors:** Hajar Faal, Linnea R. Meier, Isaiah J. Canlas, Kelly Murman, Matthew Wallace, Daniel Carrillo, Miriam F. Cooperband

**Affiliations:** ^1^ Forest Pest Methods Laboratory, USDA‐APHIS‐PPQ‐S&T, Buzzards Bay, MA, United States; ^2^ Tropical Research and Education Center, University of Florida, Homestead, FL, United States; ^3^ Biology Department, East Stroudsburg University, East Stroudsburg, PA, United States

**Keywords:** semiochemicals, pheromones, kairomones, honeydew, aggregation

## Abstract

The spotted lanternfly (SLF), *Lycorma delicatula* (Hemiptera: Fulgoridae), is a generalist phloem feeder that produces copious amounts of honeydew, which in turn coats the understory. These insects form large aggregations covering the trunks of some trees, while similar trees nearby mysteriously seem unattractive. We investigated whether volatiles from SLF honeydew are attractive to conspecifics by collecting honeydew from the field and testing it for SLF attraction in a two-choice olfactometer. We found that honeydew excreted by adult male SLF was significantly attractive to male SLF, but not female SLF. Although the honeydew excreted by adult female SLF did not significantly attract male or female SLF, both sexes showed a positive trend towards attraction in response to female honeydew in the olfactometer. Analysis of the headspace volatiles of honeydew was conducted, and numerous semiochemicals were identified. Five of which, 2-heptanone, 2-octanone, 2-nonanone, benzyl acetate, and 1-nonanol, were tested in two-choice behavioral assays against a blank control. Benzyl acetate and 2-octanone were attractive to both sexes, whereas 2-heptanone was only attractive to males, and 2-nonanone only to females. The remaining compound, 1-nonanol, repelled females, but not males. Although honeydew has been reported as a source of kairomones for some natural enemies, this may be the first report of sex-specific attractants for conspecific insects found in the honeydew volatiles of a planthopper.

## Introduction

The spotted lanternfly, *Lycorma delicatula*, (Hemiptera: Fulgoridae) (SLF) is an invasive species in Northeastern United States. Although their preferred host plant is *Ailanthus altissima* (Mill.) Swingle (Simaroubaceae) (1), they have a broad host range, including economically important species such as grapes, fruit trees and hardwood species (2). SLF causes damage by extensive phloem feeding and a large volume of honeydew secretion. This heavy feeding behavior, particularly during the adult stage, has devastated some vineyards in Pennsylvania (3). SLF has spread to numerous states and threatens agricultural, residential, and industrial areas despite the establishment of a restrictive quarantine zone in Pennsylvania and tripling applications of insecticides (4). Tools for non-insecticide control of this pest are in the early stages of development, such as the potential use of biological control agents like fungal pathogens (5), or parasitoid wasps (6). To implement any broad-scale control program, the distribution of the pest must first be determined. Therefore, our efforts have been aimed at the development of traps and semiochemical lures in order to develop survey, detection, and mass trapping tools (7–9).

Planthoppers perceive and respond to host plant volatiles (7, 8, 10), but little is known about the role of insect-produced volatiles such as pheromones in fulgorids. Recently, however, we documented evidence suggesting pheromone use may occur in SLF. Mid (during mating time) male SLF were attracted to extracts of Mid female SLF in laboratory behavioral bioassays (11). In field studies, aggregation behavior was generated in wild populations by placing groups of male or female SLF on trees in sleeves, and the sex ratio of the arriving SLF was biased toward the sex of SLF within each sleeve. Females, particularly before mating, were strongly attracted to sleeves containing female SLF, and Mid males were strongly attracted to sleeves containing Mid females. Courtship behavior was mainly observed during Mid on trees with confined females (12). Honeydew is produced by all phloem-feeding hemipteran insects, such as aphids (13), whiteflies (14), mealybugs (15), and planthoppers (16). For predators and parasitoids that attack hemipterans, volatile chemicals from honeydew are perceived as a kairomones, facilitating host habitat discovery by parasitoid wasps (17), coccinellids (18), chrysopids (19), mirid bugs (20), and flies (21, 22). Chemicals associated with honeydew may also serve as an oviposition stimulus for natural enemies (18, 23). The prospect of SLF honeydew emitting semiochemicals is clear from our observations of a variety of visiting hymenopterans that use it as a food source (24). Placing confined groups of SLF on trees generated aggregations of wild SLF in the field (12). Since their honeydew is produced in copious amounts (24), it was logical to investigate the potential role honeydew volatiles may play in the process of conspecific SLF attraction and aggregation. We hypothesized that SLF honeydew releases semiochemicals that inform other SLF about host resources, aggregations, or mates. Therefore, we sought to test whether volatiles from SLF honeydew were somehow involved in SLF attraction. Thus, this study aimed to 1) evaluate how volatiles from SLF honeydew influence SLF behavior, 2) identify any behaviorally active components and 3) define their behavioral function.

## Methods

### Timing

Developmental rates vary between year, location, and microclimate, and depend on local meteorological conditions such as degree days (25, 26). The adult stage of SLF is relatively long-lived, with eastern Pennsylvania typically seeing adult emergence in the end of July, mating in September, followed by oviposition, and finally death in late October or early November, a period spanning approximately 15-16 weeks. It is, therefore, necessary to break down the lengthy adult stage into shorter periods defined by their physiological state as it pertains to their behavior. Thus, the three time periods previously described in (7), “Early”, “Mid”, and “Late” were used. The onset of each adult phase was defined by the first field observation of its corresponding physiological state: adult emergence (Early), mating (Mid), and oviposition (Late). The calendar dates of these phases vary slightly depending on differences in latitude and climatic conditions at different field sites, and were based on the contemporary field observations at the collection sites. In 2019, start dates for adult phases were 22 July for “Early”, 8 September for “Mid”, and 22 September for “Late”.

### Field collection of honeydew and insects

**Figure 1:**
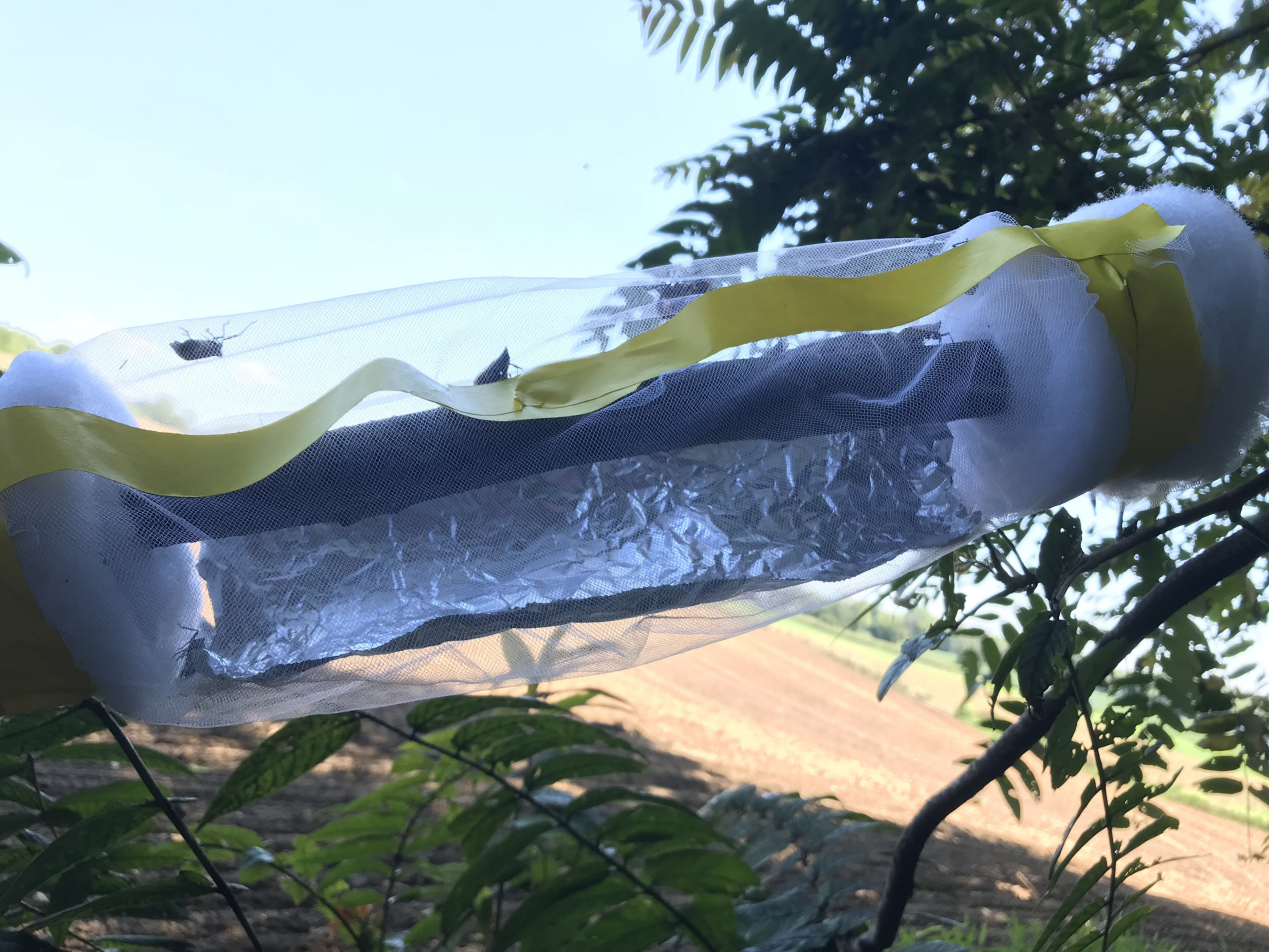
A custom mesh sleeve containing a foil “honeydew hammock” for SLF honeydew collection.

On a weekly basis, honeydew samples were collected from SLF feeding on *A. altissima* in the field in Lehigh County, PA. Woody, sun-exposed branches were carefully selected away from overhanging branches to reduce honeydew falling from above. Custom mesh sleeves (tulle, 30 cm L x 60 cm circ) were wrapped around branches (5-7 cm diam), with three layers (7 cm thick) of foam batting (Bug Barrier, Envirometrics Systems Inc., Victor, NY) at the ends to space the tulle from the branch, secured by zip ties, and closed lengthwise with lab tape (Research Products International, Mount Prospect, IL). Wearing gloves, aluminum foil (20 by 40 cm, Reynolds Consumer Products, Lake Forest, IL) was suspended like a hammock below the branch inside of each sleeve for honeydew collection (**Figure 1**). A group of either 20 male or 20 female adult SLF were placed inside each sleeve and allowed to feed and produce honeydew for 48 h. In this way, honeydew of known age, from a known number and sex of SLF, was collected on the foil. Four foil “honeydew hammocks” in sleeves were installed per week: two with males and two with females. After 48 hours, foil hammocks were removed, folded with the honeydew inside, and individually placed into single-use pre-baked oven bags which were tied tightly closed (Turkey size, Reynolds Consumer Products, Lake Forest, IL). Oven bags had been pre-baked at 150°C for 4 hours to remove volatile contaminants such as caprolactam (27). These bags containing the honeydew-laden foil were immediately placed in a cooler with dry ice. In addition, a control piece of foil which was not exposed to honeydew or SLF was handled and packaged in the same way and placed into the cooler, in case volatile compounds were inadvertently transferred to the foil during the handling and shipping process. The cooler was shipped overnight to the USDA Forest Pest Methods Laboratory (FPML) (formerly Otis Laboratory) in Buzzards Bay, Massachusetts. Each week, honeydew hammocks were set up on Monday, retrieved and shipped overnight on Wednesday, received by the FPML on Thursday morning, and used immediately upon arrival for volatile collections and behavioral bioassays (see below). This occurred weekly between 19 August and 27 September, 2019, which spanned Early, Mid, and Late phases.

Every Monday, live SLF were captured from the field and shipped overnight (as per conditions set by permits USDA P526P-15-00152 and PA PP3-0123-2015). The live insects were received on Tuesday at the FPML insect containment facility for use through Friday of the same week in behavioral bioassays and electrophysiology. There they were housed in cages (24.5 × 24.5 × 63 cm, Bugdorm, Megaview Science Co., Ltd., Taichung City, Taiwan) in an environmental chamber at 25 C with 16:8 L:D, and fed freshly cut *A. altissima* branches maintained in hydroponic solution (Maxigrow, GenyHydro Inc., Sebastopol, CA, prepared according to label).

### Honeydew standardizing and headspace volatile collections

It was necessary to standardize the amount of honeydew used in behavioral bioassays using filter papers. Thus, the amount of honeydew that could saturate a 5 mm x 10 mm piece of filter paper was used in bioassays. All handling was done wearing gloves. Prior to use, a filter paper (Whatman, grade 1, 12.5 cm circles, China) was cut into 5 mm x 10 mm rectangles and washed by soaking them in a beaker containing 100 ml of hexane for 5 min and allowed to air dry on clean foil. Upon arrival of honeydew samples in the laboratory, one at a time, each frozen foil honeydew hammock was removed from dry ice and its oven bag, unfolded, and the foil was wiped with a pre-washed 5 mm x 10 mm piece of filter paper held by a clean pair of forceps until it became saturated. This filter paper was immediately tested for attraction in the y-plate olfactometer in a different room (described below). Additional filter papers were used to collect as much of the remaining honeydew as possible from the foil using the same technique until there was none left. These remaining filter papers, laden with crude honeydew, were used to collect and analyze the volatile headspace components of the honeydew. They were placed inside a clean glass Pasteur pipette, and the wide end of the pipette was covered with aluminum foil. An absorbent solid phase microextraction fiber (SPME, 23 ga 100 μm polydimethylsiloxane, Supelco Inc., Bellfonte, PA) was selected because of its sensitivity in detecting minute amounts of volatile molecules such as insect semiochemicals, whereas preliminary attempts using other volatile collection techniques lacked such sensitivity. The SPME fiber was inserted through the narrow end of the pipette and was exposed to the headspace of the honeydew-laden filter papers for 2 h at 22°C. This process was repeated for each foil hammock and the control.

### Analysis of honeydew volatiles

Each SPME fiber was desorbed in the injection port of an Agilent 7890B gas chromatograph (GC) coupled with an Agilent 5977A mass spectrometer (MS) (EI mode, 70 eV with a scanning range of 40.0–450.0 m/z), using a DB-5MS capillary column (Agilent, 30 m×0.25 mm i.d., 0.25 µm film thickness) in splitless mode, with helium carrier gas at constant flow rate of 1 ml/min. The injection port temperature was 280°C, and the oven temperature was held at 40°C for 1 min, ramped at 10°C/min to 300°C, then held for 25 min. Tentative identifications of the honeydew volatile components were made by comparing mass spectra with those in the mass spectral library database (Enhanced ChemStation, MSD Chemstation, Data Analysis software vF.01.00.1903, and NIST, v11, Agilent Technologies, Santa Clara, CA). Close matches were confirmed by obtaining and injecting authentic standards and comparing their Kovat’s indexes (KI), retention times, and mass spectra to ensure they matched. Compounds that were also present in controls are not reported. Peak areas representing the total ion abundance for each peak were used to calculate the percent (ratio) of each identified compound over all SPME volatile collections combined for each sex (4 Early, 2 Mid, and 1 Late). The sum of peak areas for each compound was divided by the total sum of all 13 compounds for males and for females to calculate ratios.

### Antennal responses to volatiles

Gas chromatography coupled with electroantennographic detection (GC-EAD) is a common electrophysiological technique used to determine which compounds in a natural volatile collection can be detected by an insect antenna (28). However, the quantity of volatile material collected from honeydew headspace was not enough for use in GC-EAD, since, compared to known amounts of injected standards, we estimate that the average peak size of honeydew headspace volatile compounds collected by SPME fibers was approximately 8 ng. Instead, antennal responses to synthetic standards of identified components were recorded using an Agilent 6890 GC, fitted with an HP-5MS column (30 m × 0.320 mm I.D. × 0.25 μm film, Agilent Technologies, Inc., Santa Clara, CA, USA) in splitless mode. The injector and programmed temperatures were the same as those described for the GC-MS. At the end of the GC column, effluent was split 1:1 (glass Y-connector, Restek, Corp., Bellefonte, PA), with half carried to a flame ionization detector (FID) at 250°C, and the other half carried out of the GC *via* a temperature-controlled arm (Syntech Temperature Controller, Kirchzarten, Germany) at 150°C, and delivered into an L-shaped glass odor delivery tube (11 mm diam.), which delivered the effluent over the antenna. Charcoal-filtered, humidified air passed through the odor delivery tube at 0.3 L/min.

An SLF head was mounted onto a ground electrode in the form of a custom-pulled glass capillary filled with Ringer’s solution (8). Adult SLF have soft and fleshy antennae (29) which collapse when the integument is penetrated, hindering early attempts at GC-EAD. Therefore, the apical tip of the arista was removed with a razor blade, and brought into contact with the glass capillary recording electrode, such that the remaining portion of the arista was enveloped in the electrode. Electrodes were positioned using custom micromanipulators (Signatone Corp., Gilroy CA, USA) secured magnetically to a steel platform (Syntech, Kirchzarten, Germany). Antennal signals were amplified using a Dam 50 differential amplifier (World Precision Instruments, Sarasota, FL, USA), passed through Hum Bug 50/60 Hz noise elimination (Quest Scientific, North Vancouver, BC, Canada), and integrated with a two-channel signal acquisition interface (IDAC-2, Syntech, Hilversum, The Netherlands). Data were collected and analyzed using GCEAD/2014 software (Syntech, Version 1.2.5, Kirchzarten, Germany). The electrophysiological activity of both male and female antennae to synthetic compounds was determined by injecting 100 ng/ul of each compound, delivering 50 ng to the antenna and 50 ng to the FID. All synthetic compounds were manufactured by Sigma-Aldrich, Inc. (St. Louis, MO), except (*Z*)-3-nonenyl acetate which was manufactured by Bedoukian Research, Inc. (Danbury, CT).

### Behavioral bioassays

The responses of male and female SLF when presented with a choice between a volatile stimulus and no stimulus (control arm) was evaluated using custom Teflon^®^ Y-plate dual-choice olfactometers [**Supplementary File**; for descriptions, see (7, 8, 30)]. Stimuli being evaluated were either (1) a honeydew-laden filter paper, or (2) 1 mg of synthetic compound. Each Y-plate was 28.6 cm long x 21.6 cm wide and 3.8 cm tall, with a channel 5.1 cm wide cut in the shape of a Y, with the choices at a 90° angle from each other. A disposable sheet of clear acetate (Apollo, Lincolnshire, IL) was affixed to the top and bottom of the plate using electrode gel (Spectra 360, Fairfield, NJ) and served as the ceiling and floor of the bioassay, and were discarded at the end of each session. Filtered, humidified air flowed through the olfactometer at 24 cm/s. Prior to their use in the olfactometer, SLF were allowed to acclimate individually inside release cages at 25°C for 30-60 min in the walk-in environmental chamber where bioassays were to be conducted. Each session of bioassays started with a newly cleaned Y-plate bioassay apparatus with all new disposable parts. At the beginning of each session, five SLF were tested individually without volatile stimuli to ensure there was no contamination or other bias in the olfactometer. In addition, dedicated control sessions were conducted using the identical protocols used for semiochemical testing, but without chemical stimuli, in order to document the baseline activity for SLF males and females under these conditions. Each insect was individually released and allowed three min to make a choice, which occurred when the insect walked halfway up one of the two arms of the olfactometer. Insects that did not make a choice in three min were counted as non-responders. Each bioassay session, evaluating a particular choice of treatment and control, tested up to 20 individual SLF composed equally of males and females in alternating order, ensuring that both sexes were offered exactly the same stimuli. The next session used a clean Y-plate, tested five more individual SLF without stimuli, then tested the stimulus and control with directions reversed. After each session, Y-plates and parts were washed with Alconox and ethanol 95%, dried overnight, and disposable parts were discarded and replaced. If the five control insects were found to have a bias (more than 1 response in either direction), that Y-plate was immediately replaced with a clean Y-plate, and the biased Y-plate was cleaned again before use.

In bioassays testing standardized honeydew-laden filter paper (described above) for attraction, a single piece of hexane-cleaned filter paper was placed in the upwind section of one arm of the olfactometer as a control, and the single (5 mm x 10 mm) piece of filter paper laden with honeydew was placed in same position of the other arm. In this experiment, each week consisted of two sessions: one testing 10 males and 10 females, alternating, to honeydew produced by males, and the other testing 10 males and 10 females, alternating, to honeydew produced by females. For each of these four tests, behavioral data was collected over four weeks (3 Early and 1 Mid).

In behavioral assays with synthetic compounds, each upwind arm received either the synthetic compound in an open microcentrifuge tube, or an empty microcentrifuge tube control (7, 8). Each synthetic compound was tested using 1 mg of neat material (Sigma Aldrich, St. Louis, MO), and all insects used were Early adults, except for an additional test of 1-nonanol using Mid adults. The frequency and direction of choice was compared using a Chi Square test, where significance at α=0.05 was reached when the G-statistic reached 3.841 or above (31, 32).

## Results

### Analysis of honeydew volatiles

GC-MS analyses of SLF honeydew volatiles revealed the presence of four ketones, six esters, and three alcohols, all of which existed in both sexes but at different ratios (**Table 1**). Two compounds in male honeydew occurred at ratios over 1.5 times higher than in female honeydew: isoamyl acetate and nonyl acetate. Conversely, the ratios of five compounds were over 1.5 times higher in females than in males: 2-heptanone, 2-nonanone, 2-phenyl ethanol, 2-ethylhexyl acetate, and 2-undecanone. The ratios of 2-octanone, benzyl acetate, 1-nonanol, and (*Z*)-3-nonenyl acetate, *n*-decyl acetate, and 1-dodecanol were similar in the honeydew volatiles of both sexes (**Table 1**). In GC-EAD analyses, all of these produced antennal responses in both SLF males and females (**Table 1**). Due to limitations in time and insects, only the first five compounds found to have antennal activity in preliminary EAD recordings were tested in behavioral assays: 2-heptanone, 2-octanone, 2-nonanone, benzyl acetate, and 1-nonanol (**Table 1**).

**Table 1:** A summary of the compounds found in the honeydew headspace volatiles collected from male and female spotted lanternflies, *Lycorma delicatula*, between 19 August and 27 September, 2019.

Compound	Relative percent ♂ ± SE (n=6)	Relative percent ♀ ± SE (n=7)	Ratios ♂ : ♀	Behaviorally Active^1^	Antennally Active	Retention index
isoamyl acetate	29.5 ± 5.9	17.1 ± 3.4	1.7: 1	–	M, F	875
2-heptanone	0.03 ± 0.2	0.1 ± 3.9	1: 3.6	Y	M, F	887
2-octanone	5.9 ± 5.3	4.8 ± 4.1	1.2: 1	Y	M, F	989
2-nonanone	3.0 ± 4.3	7.0 ± 5.3	1: 2.4	Y	M, F	1088
2-phenyl ethanol	12.2 ± 2.9	19.6 ± 13.3	1: 1.6	–	M, F	1108
2-ethylhexyl acetate	2.5 ± 1.6	4.5 ± 3.8	1: 1.8	–	M, F	1146
benzyl acetate	24.5 ± 12.3	22.8 ± 4.9	1.1: 1	Y	M, F	1150
1-nonanol	4.9 ± 5.4	5.9 ± 7.8	1: 1.2	Y	M, F	1170
2-undecanone	1.2 ± 1.8	5.0 ± 3.9	1: 4.1	–	M, F	1290
(*Z*)-3-nonenyl acetate	4.5 ± 1.0	4.3 ± 0.9	1: 1	–	M, F	1290
nonyl acetate	9.1 ± 2.8	5.4 ± 1.2	1.7: 1	–	M, F	1307
n-decyl acetate	2.7 ± 0.6	3.4 ± 0.7	1: 1.3	–	M, F	1407
1-dodecanol	1.7 ± 0.4	2.1 ± 0.5	1: 1.2	–	M, F	1470

^1^Behaviorally active components are indicated (Y). Minus signs “-” denote the compounds that were not tested in behavioral bioassays.

Antennal responses to synthetic compounds were recorded from both males (M) and females (F).

### Behavioral assays

**Figure 2:**
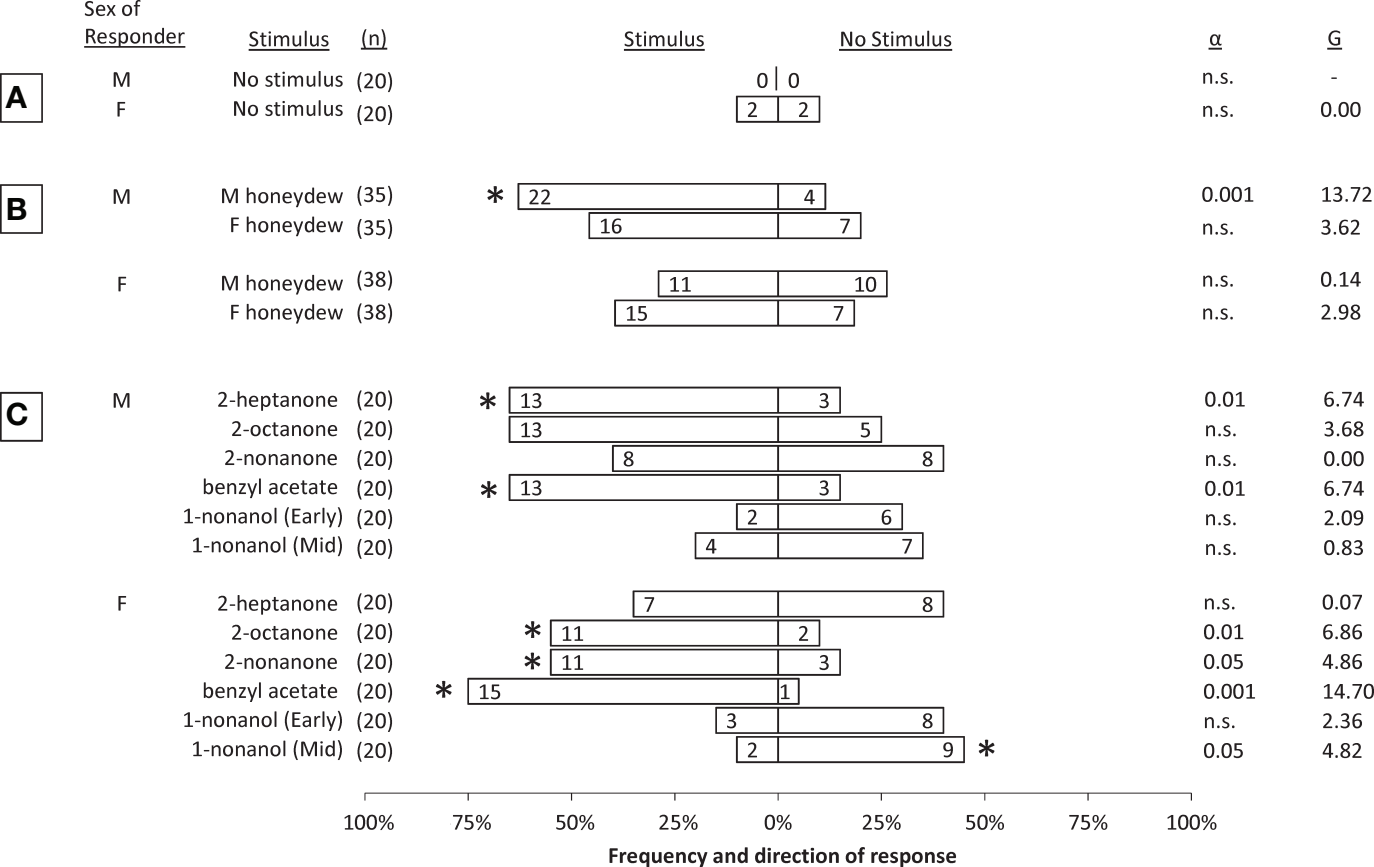
Choices made by male and female spotted lanternflies, *Lycorma delicatula*, in dual-choice bioassays comparing no stimulus to: **(A)** no stimulus (controls); **(B)** volatiles emitted from honeydew excreted either by male (M) or female (F) conspecifics, and **(C)** synthetic compounds (1 mg) found in honeydew volatiles. All tests were conducted using Early adults except where indicated. The numbers inside the bars indicate the numbers of insects that responded to the respective choice within 3 min. The number of insects tested (n) (including non-responders) are shown for each test. Asterisks represent a significant deviation from expected frequencies between two choices with critical α levels and G-statistics provided (Chi Square test). Alpha below 0.05 is not significant (n.s.).

Control sessions showed that alternating male and female SLF tested in the olfactometer had low response rates and no directional bias (**Figure 2A**). Honeydew volatiles from either male or female SLF produced different levels of attraction of males or females, compared to the control arm in the y-plate olfactometer. By testing 5 mm x 10 mm filter papers saturated with honeydew, potential unknown differences in amount of honeydew production between males (which are smaller) and females (which are larger) can be ruled out. Thus, differences in male and female attraction likely can be ascribed to differences in composition between the honeydew produced by males and females. Male SLF were significantly attracted to the volatiles of honeydew excreted by male SLF, with an overall response rate of 74% (G=13.72, α=0.001, df=1, n=35). Female SLF were not attracted to male honeydew volatiles. Neither sex was attracted significantly to female honeydew volatiles, however, both males and females showed a trend towards attraction to female honeydew volatiles that approached significance (**Figure 2B**).

In behavioral assays with synthetic compounds, benzyl acetate was significantly attractive to both sexes during Early, 2-heptanone was significantly attractive only to Early males, 2-octanone was significantly attractive to Early females but males trended towards it, and 2-nonanone was significantly attractive only to Early females. Conversely, 1-nonanol trended in the opposite direction for Early males and females, and had a significant repellent effect on Mid females but not Mid males (**Figure 2C**).

## Discussion

In the current study we described the behavioral function and volatile profiles of honeydew derived from adult male and female SLF. Adult males, but not females, were significantly attracted to male honeydew volatiles. A trend of attraction by both male and female SLF to honeydew volatiles derived from females suggests that female honeydew volatiles may have shown attraction with more replication or with more material in the olfactometer. Interestingly, the fact that male honeydew volatiles attracted only male SLF in bioassays aligns with field results found by Cooperband and Murman (12). In that study, wild male SLF were attracted to host trees with sleeves containing confined aggregations of males, resulting in a significantly male-skewed wild sex ratio on those trees. Conversely, significantly more female-biased wild SLF sex ratios occurred on trees that had confined female aggregations (12). Strongly skewed sex ratios with either male or female bias on different trees, or at different times in the season, have been documented in SLF (12, 33, 34). Thus, a potential mechanism for the observed phenomenon of extreme sex ratio bias in SLF field aggregations is presented here.

Although all 13 compounds described here from SLF honeydew headspace volatiles were eventually found to elicit antennal responses, technical challenges in initially developing EAD capabilities with adult SLF antennae hampered the beginning of this study. Limitations in time and insects led us to select only the first five compounds that were found to be antennally active to test for attraction. The issues were resolved in a subsequent year, and EAD was conducted again on all compounds, which were all found to elicit antennal responses from both male and female SLF. Unfortunately, conducting behavioral bioassays on the remaining compounds was not possible due to the time and logistical constraints when working with this univoltine insect.

Volatiles from SLF honeydew headspace were identified as ketones, esters, and alcohols. Similar chemical profiles were documented from the honeydew headspace volatiles of both sexes, but they occurred in different ratios. However, those ratios were not fixed over time. This study did not seek to evaluate seasonal changes in chemical ratios. Instead, we reported the average ratios taken over the season from Early, Mid, and Late adult SLF. Benzyl acetate attracted both Early males and females in the y-plate olfactometer, whereas 2-heptanone attracted only Early males, and conversely, 2-octanone and 2-nonanone attracted only Early females. One identified compound, 1-nonanol, showed a significant repellent effect on Mid females and no effect on Mid males. Preference differences between males and females for specific ratios of the same chemicals might explain why male SLF were attracted to honeydew derived from males, but females were not. The fact that SLF produce large quantities of honeydew that can be collected, and the sensitivity of the SPME fibers and the GC-MS, facilitated our ability to collect and detect the presence of minute quantities of volatile compounds. With an average peak containing about 8 ng of material, however, we cannot rule out the possibility of a sex-specific compound that may be present below our level of detection.

Studies in other hemipterans have demonstrated the importance of volatiles from honeydew in attracting natural enemies. Honeydew volatiles described for several species include hydrocarbons, disulfides, ketones, alcohols, aldehydes, carboxylic acids, a pyrazine, and a monoterpene (14, 18, 21). Most studies on honeydew were focused on carbohydrate contents as a food source for natural enemies and ants (35). Conspecific and sex-specific attraction to honeydew has been documented to occur in psyllids, in which only males were attracted to conspecific honeydew, but the responsible compounds were not characterized (36). To our knowledge, this is the first evidence of attraction to conspecific honeydew volatiles in a planthopper.

It is well documented that SLF honeydew accumulates and thickly coats the trunks and bases of *A. altissima* trees, and may become white and frothy over time when SLF densities are high (24) which can also produce a strong fermentation odor (MC, pers. obs.). The honeydew in this study accumulated for only two days on a clean foil surface. Although beyond the scope of the current study, we should not ignore the potential role of microbes dwelling in hemipteran honeydew as a source of volatiles which may act as semiochemicals (37). Several studies isolated bacteria from hemipteran honeydew (38, 39), the volatiles of which acted as kairomones for natural enemies (21, 37) or mosquitoes (21). A wide range of chemicals have been described from bacterial volatiles, including alcohols, aldehydes, carboxylic acids, esters, hydrocarbons, and ketones (40–42), but none were the same compounds we collected from SLF honeydew headspace.

All of the compounds found in SLF honeydew are known to occur in both plants (43–52) and insects (53–65). The five compounds tested for attraction all serve as pheromone components for species across multiple insect orders. For example, benzyl acetate was found in pheromones of bees (53) and bed bugs (54). The current study is the first report, to our knowledge, of a planthopper species attracted to benzyl acetate. In ants, 2-heptanone has been reported as part of an alarm pheromone (55). We found sexual differences in SLF attraction to 2-heptanone and 2-nonanone (**Figure 2**), and interestingly, such sexual differences are present in other insects as well (56, 66). For example, 2-octanone, one of numerous compounds found in the excreta of mixed sex groups of bedbugs *Cimex hemipterus*, produced a positive attraction index in only male bedbugs (56). The compound 2-nonanone has been reported as an aggregation pheromone (57), sex pheromone component (58), and alarm pheromone component in ants (55). In a fly, 1-nonanol was suggested as a female attractant (67).

There are numerous avenues one could pursue for additional research, for instance, investigating whether the host plant species being fed upon alters the volatile profile and attractiveness of honeydew (68). Volatile and sugar profiles of hemipteran honeydew may vary with different host plants (68). In the current study, SLF honeydew was collected while they were feeding on *A. altissima.* SLF have a wide range of host plants with different volatile profiles (1, 8), but their host range narrows as they develop, and adults accumulate on *A. altissima* (69, 70). Examining the volatile profiles and attractiveness of SLF honeydew produced while feeding on other host plants could be a revealing way to study their host plant relationships and may help narrow down important semiochemicals. Our bioassays used 1 mg lures, a dose previously used to test SLF attraction to host plant volatiles, or kairomones (8), which typically occur in larger amounts than pheromones. Dose-response studies could reveal whether the compounds are behaviorally active at the nanogram range or lower, which is the range expected for a pheromone (71). In addition, synthetic blends of honeydew volatiles in sex-specific ratios should be tested for attraction of males and females.

In an effort to determine how SLF locate each other from a distance for purposes of mating or the formation of aggregations, this study evaluated SLF honeydew volatiles as a possible mechanism for conspecific attraction, and described the components of headspace volatiles from SLF honeydew. All honeydew compounds elicited antennal responses from male and female SLF adults, and the behavioral function for male and female SLF of five of those compounds individually was described. Our results introduce a potential new mechanism for SLF, and perhaps other honeydew producers, to locate conspecifics in response to semiochemical cues emitted from their own honeydew. This mechanism also may be involved in driving the male- or female- skewed SLF sex ratios observed to naturally occur on different trees at specific times in adult development (12, 33). Complete behavioral testing of each of the remaining compounds as well as synthetic blends would help to fully understand this system. In addition, dose response testing could improve our understanding of behavioral function, as some compounds may be attractive at low doses and repellent at high doses.

## Data availability statement

The raw data supporting the conclusions of this article will be made available by the authors, without undue reservation.

## Author contributions

MC secured funding, conceived and designed experiments, analyzed data, and wrote the manuscript; HF analyzed GCMS files, conducted GCEAD, and wrote the manuscript; LM processed honeydew samples, collected volatiles, prepared and injected samples on GCMS, and conducted preliminary GCEAD and preliminary analysis of honeydew GCMS files; IC conducted dual-choice bioassays; KM collected honeydew and insects, oversaw all field components, and edited the manuscript; MW supervised personnel, facilitated research, and edited manuscript; DC supervised personnel, facilitated research, and edited manuscript. All authors contributed to the article and approved the submitted version.

## Funding

Funding for this work came from the Plant Protection Act, Section 7721, for project 3.0386 in 2019 which funded the work of the FPML and cooperative agreements AP19PPQS&T00C028, AP19PPQS&T00C036, and AP19PPQS&T00C016; and project 3.0792.03 in 2021 which funded agreement AP21PPQS&T00C065.

## Acknowledgments

We thank Stefani Cannon, Kyle Kaye, Kerry Handelong, Zachary DeSantis, and Daniel Rinkenberg for their essential help collecting SLF and honeydew in the field in 2019, Sam Stella for laboratory support and maintaining insect colonies, and Annie Ray for providing laboratory support. We also thank the property owners who allowed us to collect SLF and honeydew on their properties. We extend gratitude to cooperators University of Florida, East Stroudsburg University, and Xavier University, and our funding source Plant Protection Act, Section 7721, for project 3.0386 in 2019 which funded the work of the FPML and cooperative agreements AP19PPQS&T00C028, AP19PPQS&T00C036, and AP19PPQS&T00C016; and project 3.0792.03 in 2021 which funded agreement AP21PPQS&T00C065. This material was made possible, in part, by a Cooperative Agreement from the United States Department of Agriculture’s Animal and Plant Health Inspection Service (APHIS). It may not necessarily express APHIS’ views.

## Conflict of interest

The authors declare that the research was conducted in the absence of any commercial or financial relationships that could be construed as a potential conflict of interest.

## Publisher’s note

All claims expressed in this article are solely those of the authors and do not necessarily represent those of their affiliated organizations, or those of the publisher, the editors and the reviewers. Any product that may be evaluated in this article, or claim that may be made by its manufacturer, is not guaranteed or endorsed by the publisher.

## Supplementary material

Supplementary Figure 1 Photograph of a Y-plate.
