# Supplementary figures and images for: Volatiles from male honeydew excretions attract conspecific male spotted lanternflies, Lycorma delicatula (Hemiptera: Fulgoridae)
Source: Front Insect Sci. 2022 Sep 27;2:982965. doi: 10.3389/finsc.2022.982965 (PMC10926466; doi:10.3389/finsc.2022.982965)

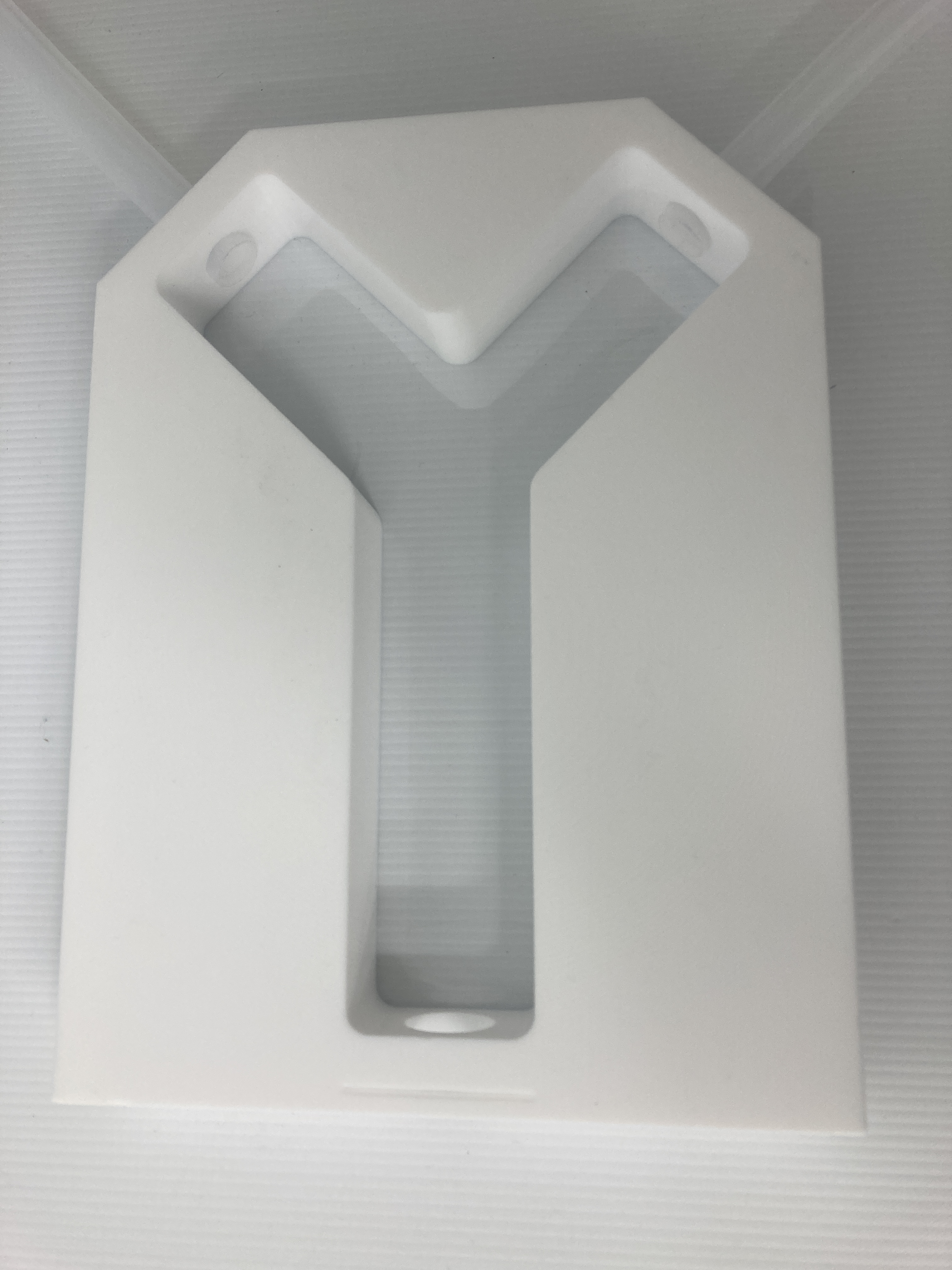

Supplement: Supplementary Figure 1 [file Image_1.jpeg]
